# Assessing Links Between Alcohol Exposure and Firearm Violence: A Scoping Review Update

**DOI:** 10.35946/arcr.v45.1.01

**Published:** 2025-01-10

**Authors:** Ellicott C. Matthay, Ariana N. Gobaud, Charles C. Branas, Katherine M. Keyes, Brita Roy, Magdalena Cerdá

**Affiliations:** 1 Center for Opioid Epidemiology and Policy, Division of Epidemiology, Department of Population Health, New York University Grossman School of Medicine, New York, New York; 2 Department of Epidemiology, Columbia Mailman School of Public Health, New York, New York; 3 Section for Health Equity, Division of Health and Behavior, Department of Population Health, New York University Grossman School of Medicine, New York, New York; 4 Division of General Internal Medicine and Clinical Innovation, Department of Medicine, New York University Grossman School of Medicine, New York, New York

**Keywords:** alcohol, firearm, gun ownership, gun possession, injury, homicide, suicide

## Abstract

**BACKGROUND:**

Firearm violence remains a leading cause of death and injury in the United States. Prior research supports that alcohol exposures, including individual-level alcohol use and alcohol control policies, are modifiable risk factors for firearm violence, yet additional research is needed to support prevention efforts.

**OBJECTIVES:**

This scoping review aims to update a prior 2016 systematic review on the links between alcohol exposure and firearm violence to examine whether current studies indicate causal links between alcohol use, alcohol interventions, and firearm violence-related outcomes.

**ELIGIBILITY CRITERIA:**

Following the Preferred Reporting Items for Systematic Reviews and Meta-Analyses Extension for Scoping Reviews (PRISMA-ScR) guidelines, a comprehensive search of published studies was conducted, replicating the search strategy of the prior review but focusing on studies published since 2015. The review included published studies of humans, conducted in general populations of any age, gender, or racial/ethnic group, that examined the relationship between an alcohol-related exposure and an outcome involving firearm violence or risks for firearm violence. Excluded were small studies restricted to special populations, forensic or other technical studies, non-original research articles such as reviews, and studies that relied solely on descriptive statistics or did not adjust for confounders.

**SOURCES OF EVIDENCE:**

The review included published studies indexed in PubMed, Web of Science, and Scopus. Eligible articles were published on or after January 1, 2015. The latest search was conducted on December 15, 2023.

**CHARTING METHODS:**

Using a structured data collection instrument, data were extracted on the characteristics of each study, including the dimension of alcohol exposure, the dimension of firearm violence, study population, study design, statistical analysis, source of funding, main findings, and whether effect measure modification was assessed and, if so, along what dimensions. Two authors independently conducted title/abstract screening, full-text screening, and data extraction until achieving 95% agreement, with discrepancies resolved through discussion.

**RESULTS:**

The search yielded 797 studies. Of these, 754 were excluded and 43 met the final inclusion criteria. Studies addressed a range of alcohol exposures and firearm violence-related outcomes, primarily with cross-sectional study designs; 40% considered effect measure modification by any population characteristic. Findings from the 21 studies examining the relationship of individual-level alcohol use or alcohol use disorder (AUD) with firearm ownership, access, unsafe storage, or carrying indicated a strong and consistent positive association. Seven studies examined associations of individual-level alcohol use or AUD with firearm injury or death; these also indicated a pattern of positive associations, but the magnitude and precision of the estimates varied. Eight studies examined the impact of neighborhood proximity or density of alcohol outlets and found mixed results that were context- and study design-dependent. Two studies linked prior alcohol-related offenses to increased risk of firearm suicide and perpetration of violent firearm crimes among a large cohort of people who purchased handguns, and two studies linked policies prohibiting firearm access among individuals with a history of alcohol-related offenses to reductions in firearm homicide and suicide. Finally, four studies examined alcohol control policies and found that greater restrictiveness was generally associated with reductions in firearm homicide or firearm suicide.

**CONCLUSIONS:**

Findings from this scoping review continue to support a causal relationship between alcohol exposures and firearm violence that extends beyond acute alcohol use to include AUD and alcohol-related policies. Policies controlling the availability of alcohol and prohibiting firearm access among individuals with alcohol-related offense histories show promise for the prevention of firearm violence. Additional research examining differential impacts by population subgroup, alcohol use among perpetrators of firearm violence, policies restricting alcohol outlet density, and randomized or quasi-experimental study designs with longitudinal follow-up would further support inferences to inform prevention efforts.

## Rationale

Firearm violence, including self-directed and interpersonal violence, remains a leading cause of death and injury in the United States. From 2014 to 2021, firearm homicide rates among Americans rose an alarming 83%, peaking at the highest levels seen in more than 20 years.^1^ In the United States, firearms are the predominant means of suicide death, and firearm suicide rates increased 40% from 2006 to 2021.^1^–^3^ In 2021, nearly 21,000 people in the United States were victims of firearm homicides, and more than 26,000 completed suicide with a firearm.^1^ Firearm violence-related risks also encompass firearm crime victimization and perpetration (e.g., robbery with a firearm), as well as behaviors such as firearm ownership, storage, and carrying.^4^,^5^ Firearm violence and related risks have evolved concurrently with shifting social and economic conditions, including the COVID-19 pandemic and related economic crises, political division, and surges in firearm purchases.^6^ Given these worrisome changes, researchers and decision-makers are renewing attention to the causes of and solutions to firearm violence. Although firearm policies are essential, they face political opposition and implementation challenges.^7^–^9^ Additional intervention points are needed.

Existing evidence suggests that alcohol use is an important modifiable risk factor for firearm violence, and programs and policies targeting alcohol use may offer opportunities for both short-term and long-term prevention of firearm violence.^10^–^12^ Alcohol use increases aggression and violent behavior, alters judgment, and boosts impulsivity, each of which may increase risk for firearm self-harm, unintentional injury, assault perpetration, or assault victimization.^13^–^21^ In places where alcohol is consumed and firearms are present, altercations may be more likely to result in firearm injury.^22^ Alcohol misuse is more common among people who own firearms than among the general population of the United States,^22^,^23^ and an estimated 11.7 million people in the United States who own firearms binge drink.^23^ (See the Results section for definitions of levels of alcohol use.) Substantial research has documented associations between alcohol use—particularly alcohol misuse, such as heavy drinking—and firearm ownership, access, carrying, or use.^19^,^22^–^32^ In turn, firearm ownership and carrying are associated with firearm injuries and crimes.^33^–^40^ Among U.S. firearm homicide decedents, 30% drank heavily immediately prior to their death, and among U.S. firearm suicide decedents, 25% drank heavily immediately prior to their death.^22^

A 2016 systematic review and meta-analysis on alcohol use and firearm violence by Branas and colleagues found that observational studies involving multivariate adjustment supported a relationship between alcohol use and firearm violence; however, the literature lacked experimental and quasi-experimental studies that rigorously determined whether this relationship was causal.^22^ Additionally, that review found almost no studies assessing the impacts of programs or policies that target alcohol use (e.g., reducing population-level alcohol availability by capping alcohol outlet density, prohibiting persons with specific alcohol-related offenses from owning a firearm) on firearm violence outcomes. An updated review therefore is needed to determine if any of these critical research gaps have been addressed. A traditional systematic review is well-suited for examining questions about a single, specific exposure or treatment. Given the diversity of relevant alcohol-related exposures, firearm-related outcomes, and corresponding analytic approaches, a scoping review is most appropriate to describe the landscape of current research in this area.

## Objectives

This scoping review examined the research on alcohol exposure and firearm violence that has been published since Branas et al.’s 2016 review. It focused specifically on assessing whether studies published since the 2016 review have established the answers to two questions: (1) Etiology: Does alcohol use cause firearm violence or firearm violence-related risks? (2) Intervention: Are there any interventions targeting alcohol use, alcohol availability, or other alcohol-related factors that effectively prevent firearm violence or firearm violence-related risks? The analyses considered alcohol-related exposures and interventions at all socioecological levels, including the individual, neighborhood, and political jurisdiction, as well as multiple dimensions of firearm violence-related risks, including fatal and nonfatal injuries; crime perpetration and victimization; and behaviors that affect the risk for firearm violence perpetration or victimization, including firearm ownership, safe storage, and carrying.^4^,^5^ Because policies often differ in their impacts on distinct population subgroups,^41^,^42^ the review further assessed whether the research considered potential unequal impacts on different population subgroups. Finally, future research that may help to rigorously determine what works to prevent firearm violence is discussed.

## Methods

### Protocol and Registration

This scoping review followed the Preferred Reporting Items for Systematic Reviews and Meta-Analyses Extension for Scoping Reviews (PRISMA-ScR).^43^ The protocol for this study was published in advance with the Open Science Framework.^44^

### Eligibility Criteria

The review included studies of humans, conducted in general populations of any age, gender, or racial/ethnic group, that examined the relationship between an alcohol-related exposure and an outcome involving firearm violence or risks for firearm violence, and that were published between January 1, 2015, and December 15, 2023. The exclusion and inclusion criteria were identical to those of the review by Branas et al.,^22^ with three exceptions. First, the current review excluded studies that characterized the proportion of firearm suicide or homicide decedents who had consumed alcohol or were acutely intoxicated at the time of death. Second, it excluded studies that characterized the proportion of firearm owners who drank alcohol or engaged in binge drinking. This review omitted these studies because they relied on descriptive statistics and lacked an appropriate comparison group with multivariate adjustment, making it difficult to reach conclusions about the causal link between alcohol exposures and risks for firearm violence. Third, the current review included studies published in other languages, whereas the earlier review excluded these.

Consistent with Branas et al.,^22^ the current review excluded articles that met any of the following criteria: (1) research not on human subjects; (2) smaller studies restricted to special populations (e.g., individuals involved in murder-suicides, individuals with schizophrenia, police officers, physicians, individuals in active war or conflict situations); (3) studies that examined substance use but did not separate out alcohol; (4) studies that examined violence, crime, homicide, or suicide but did not separate out those involving firearms specifically (e.g., pooled firearm and non-firearm violence, only examined “weapons”); (5) studies that did not examine both firearms (or equivalent synonyms) and alcohol (or equivalent synonyms); (6) forensic or other technical studies; (7) non-original research articles such as reviews, meta-analyses, and editorials; (8) forensic science case series; (9) studies that did not report an adjusted measure of association between an alcohol-related measure and a firearm-related measure; (10) articles published in 2014 or prior; and (11) conference abstracts.

### Information Sources, Search, and Selection of Sources of Evidence

Three health and social sciences databases were searched for this review: PubMed, Web of Science, and Scopus. Given the goal of updating the prior review by Branas et al., this review uses search criteria that were identical to the earlier review, except that this review includes studies published in other languages.

The full electronic search strategy by database, along with the number of results retrieved for each database, is presented in Table 1. Web of Science and Scopus searches focused on the title/abstracts/keywords. The latest search was conducted on December 15, 2023. Search results were downloaded, deduplicated, and uploaded to Covidence for screening.

### Title/Abstract and Full-Text Screening

Using Covidence, authors sorted entries by author and year, and two authors (E.C.M. and A.N.G.) independently screened the first 100 titles and abstracts to assess if they warranted full-text review. The authors met to discuss discrepancies in batches of 20 articles and continued this process until they reached 95% agreement. In this phase, articles were included for full-text review if they met the inclusion criteria or if full-text review was needed to determine whether the study met the inclusion criteria. Articles passing title/abstract screening then underwent independent full-text review by the same two authors. During this phase, articles were included if they did not meet the exclusion criteria described above.

### Data Charting Process

A structured data collection instrument in Covidence was used to extract data from each study. Entries were sorted by author and year, and two authors (E.C.M. and A.N.G.) independently extracted data from the first 15 articles. The authors met to discuss discrepancies in batches of five articles and continued this process until they reached 95% agreement.

### Data Items

Specific data elements extracted from each of the articles included the title, authors, publication year, journal, dimension of alcohol (i.e., use, binge, chronic excessive, use disorder, outlets, policies), dimension of firearms (i.e., injury, type of injury, crime perpetration, crime victimization, access, ownership, storage practices, carrying, outlets, policies), study design (e.g., cohort, case-control), unit of analysis (e.g., individual, state), covariates, statistical analysis, characteristics of the study population (i.e., location, age, gender, years, sample inclusion criteria), type of measure of association (e.g., odds ratio, risk difference), direction of effect (harmful, protective, null), statistical significance, main finding, whether effect measure modification was assessed and, if so, along what dimensions, and source of funding. Authors categorized firearm injuries into the following groupings: intentional self-directed, intentional interpersonal, unintentional (interpersonal or self-directed), and undetermined intent.

### Critical Appraisal of Individual Sources of Evidence

Because of the tremendous heterogeneity in study questions, exposures, outcomes, and study designs included in this scoping review, no critical appraisal or risk-of-bias assessment for the individual studies was performed. For example, risk-of-bias assessment tools are typically specific to the type of study design (e.g., cohort, case-control) and require identifying the key threats to validity (e.g., confounding variables) that must be addressed to validly estimate a causal effect for the given exposure and outcome. However, for example, the relevant threats to validity for a case-control study examining the impacts of participants’ minute-by-minute proximity to alcohol outlets over the course of the day on risk of firearm assault victimization are completely distinct from those of a state-level cross-sectional time-series analysis of the impacts of changes in state beer excise taxes on firearm suicide rates. Therefore, authors considered study quality more broadly, based on factors such as the study design and the depth of confounder assessment. In general, studies with individual-level longitudinal follow-up and thorough confounder control were deemed to be higher quality, but this assessment was not systematic.

### Synthesis of Results

Results were organized by category of alcohol exposure operated. The scoping review results are described qualitatively, identifying major themes; the review used evidence tables to summarize findings.

## Results

### Selection of Sources of Evidence

**Figure 1:**
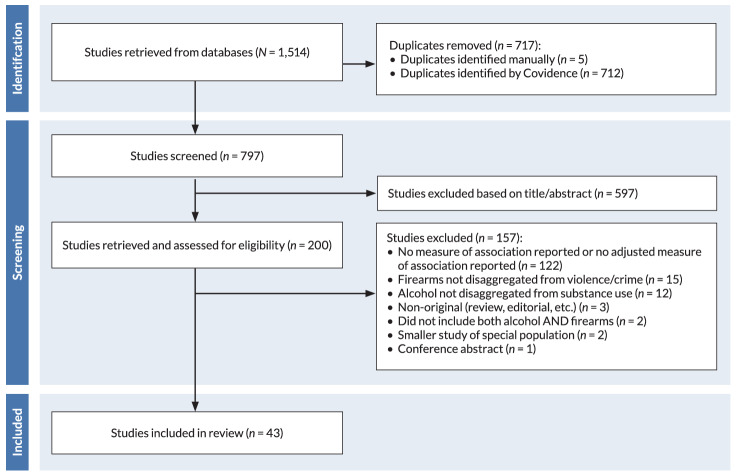
Flow diagram showing selection process for research articles on alcohol and firearm violence, 2015–2023 (PRISMA). *Note:* CINAHL, Cumulative Index to Nursing and Allied Health Literature.

The search strategy yielded 1,514 studies from PubMed, Scopus, and Web of Science. After removing duplicates, authors screened the titles and abstracts of 797 unique studies (Figure 1). Of these, 200 passed title/abstract screening. Full-text screening identified 122 studies that captured alcohol- and firearm-related measures but did not meet the specified inclusion criteria and thus were excluded. Of these 122 studies, 69 studies were excluded because they did not report any measure of association (even unadjusted) between the alcohol exposure and the firearm outcome, 25 studies did not report an adjusted measure of association between the alcohol exposure and the firearm outcome, 27 studies reported only the proportion of firearm cases involving alcohol or the proportion of alcohol cases involving firearms, and one study examined the impact of firearm policies on alcohol-related mortality rather than the reverse. An additional 15 studies were excluded because firearm involvement was not disaggregated from overall measures of violence, crime, or weapons, and 12 studies were excluded because alcohol was not disaggregated from the general category of substances or substance use. After a full-text review, the final sample included 43 studies.^31^,^45^–^86^

### Definitions of Alcohol Use

Alcohol exposure measures varied across studies. Unless otherwise specified hereafter, “alcohol use” refers to consumption of one or more alcoholic beverages or drinks over the time period designated by the study, often the past week or past 30 days. One “drink” refers to consumption of one U.S. standard serving of alcohol, corresponding to 12 ounces of regular beer, 5 ounces of wine, 1.5 ounces of distilled spirits, or 14 grams of pure alcohol. “Binge drinking” refers to consuming four or more drinks in one session for women and five or more drinks in one session for men, with the definition of a “session” varying across studies. “Heavy drinking” refers to consuming seven or more drinks per week for women and 14 or more drinks per week for men. “Chronic” or “excessive” alcohol use refers to consuming eight or more drinks in 1 week for women and 15 or more drinks in 1 week for men. “Habitual” alcohol use refers to an affirmative answer to the question “Do you consume alcoholic beverages habitually, even if only very seldom or on special occasions?”

### Comparison to the Branas et al. 2016 Review

The average number of studies published annually on alcohol exposure and firearm violence increased from 1.3 in the years covered by Branas et al.^22^ (1975 to 2014) to 4.8 in the years covered by the current review (2015 to 2023). This increase incorporates the slight differences in the inclusion/exclusion criteria between the two reviews. If the current review had used the same inclusion criteria as the 2016 review and therefore included descriptive studies and studies reporting unadjusted associations, it would have included an additional 54 studies, for an average annual study count of 10.8.

The topical areas of focus also shifted over time (Table 2). In particular, in this review, a smaller proportion of studies addressed individual alcohol use, and a greater proportion addressed alcohol use disorder (AUD), alcohol-related offenses (e.g., convictions for driving under the influence [DUI]), neighborhood proximity or density of alcohol outlets, alcohol control policies, and policies restricting access to firearms based on alcohol-related offenses.

### Characteristics of Sources of Evidence

Table 2 reports the number of studies covering the following topics of alcohol exposure: At the individual level, 21 studies examined alcohol use,^31^,^45^–^49^,^51^,^53^–^55^,^57^–^59^,^61^–^66^,^68^,^71^ eight studies examined AUD,^50^,^52^,^56^,^60^,^67^,^69^–^71^ and two studies examined alcohol-related offenses (e.g., DUI convictions).^79^,^80^ At the neighborhood level, eight studies examined alcohol outlets.^71^–^78^ At the policy level, four studies examined alcohol control policies,^81^–^84^ and two studies examined policies limiting access to firearms based on prior alcohol offenses.^85^,^86^ Of the six studies examining individual-level alcohol factors (use, AUD, offenses) and interpersonal violence firearm injury or crime, two studies^64^,^80^ examined alcohol in relation to the perpetrator whereas four studies^63^,^67^,^69^,^71^ examined alcohol use of the victim.

Appendix 1 describes the characteristics and results of the 43 studies meeting inclusion criteria. Firearm violence-related outcomes included fatal or nonfatal firearm assault injuries,^68^,^69^,^71^,^75^–^77^,^82^–^86^ fatal or nonfatal firearm self-harm injuries,^69^,^70^,^78^,^79^,^81^,^82^,^84^–^86^ firearms as a means of assault or homicide (versus other means),^67^ firearms as means of self-harm or suicide (versus other means),^65^,^66^,^81^ violent crimes involving a firearm,^72^–^74^,^80^ firearm access (have a firearm or could acquire one quickly),^31^,^47^,^48^ firearm ownership or possession,^50^–^52^ firearm purchases,^49^ firearm carrying,^55^–^59^ firearm safe storage,^51^–^54^ composite measures of firearm exposure or involvement,^46^,^60^ desire to own a firearm for protection,^45^ or other forms of firearm use (fights involving a firearm,^62^ alcohol-impaired hunting or target practice,^61^ firearm violence perpetration behaviors^64^).

In terms of study settings and populations, 16 studies examined the entire United States,^49^,^50^,^52^,^54^–^58^,^65^,^66^,^68^,^81^–^85^ eight studies focused on a single U.S. state,^51^,^53^,^67^,^70^,^77^–^80^ 17 studies focused on a single or few localities (e.g., Philadelphia),^31^,^46^–^48^,^59^–^61^,^63^,^64^,^69^,^71^–^76^,^86^ and two studies examined international settings (both in Brazil).^45^,^62^ Study populations were predominantly censuses of firearm-related outcomes (e.g., national death records, trauma registry data, or city crime records) or population-representative samples (e.g., state Behavioral Risk Factor Surveillance Surveys). Fourteen studies^31^,^46^–^48^,^52^,^59^–^61^,^63^,^64^,^69^,^73^–^75^ used selective or convenience samples, such as tailored cohort studies, and one study used simulated data.^86^ Within the chosen study setting, 12 studies examined outcomes for the population overall,^68^,^69^,^72^–^74^,^76^–^78^,^81^,^82^,^84^,^85^ 14 focused on adults (typically ages 18 years or older),^45^,^46^,^49^–^53^,^60^,^66^,^67^,^70^,^79^,^80^,^86^ 16 focused on youth or adolescents (typically between ages 10 to 24 years),^31^,^47^,^48^,^54^–^59^,^61^–^64^,^71^,^75^,^83^ and one focused on older adults (ages 50 years or older).^65^ All studies focused on outcomes among all sexes/genders, except for one study that focused on assault injuries among young Black men in Arkansas,^67^ and one study that focused on adult women involved in an HIV prevention intervention.^46^

In terms of methodology, cross-sectional study designs were the most common.^31^,^45^–^53^,^60^–^62^,^65^–^67^,^81^ Other designs included cohort,^54^,^57^–^59^,^63^,^64^,^79^,^80^ case-control,^56^,^69^–^71^,^74^,^75^,^77^,^78^ cross-sectional time series,^55^,^68^,^82^,^84^,^85^ geospatial (e.g., using the Cross-K Function statistical tool),^72^,^73^,^76^ quasi-experimental,^83^ and simulation modeling.^86^ Most studies were conducted at the individual level, but several—particularly those assessing the impacts of outlet densities or policies—were conducted at the area level, most commonly U.S. states. Only 17 studies (40%)^53^,^57^,^59^,^62^,^66^,^72^,^73^,^75^–^81^,^83^–^85^ considered effect measure modification by any population characteristic or other relevant dimension. Of these, studies examined diverse effect measure modifiers: sex or gender,^62^,^66^,^80^,^85^ age,^53^,^57^,^59^,^66^ race/ethnicity,^80^ urbanicity,^53^ socioeconomic index,^73^ time of day or week,^72^,^75^,^84^ U.S. state or other state policies,^81^,^83^ on-premise versus off-premise alcohol outlets,^76^ and other aspects of individual risk (e.g., age-sex-race combination, characteristics of the person purchasing a firearm).^77^–^79^

Of the 43 included studies, most found that greater alcohol exposure was associated with higher levels of firearm violence or related risks, or analogously that more alcohol-related restrictions were associated with lower levels of firearm violence or related risks. However, only a subset of the associations was statistically significant.^31^,^45^–^47^,^49^–^51^,^53^–^55^,^57^,^62^,^63^,^68^,^72^,^74^,^79^–^81^,^86^ Mixed results,^58^,^59^,^66^,^69^,^71^,^73^,^75^–^78^,^82^,^84^,^85^ null results (i.e., the estimated measure of association was 0 on the additive scale or 1 on the relative scale),^64^ and protective associations^31^,^60^,^63^,^67^ of alcohol exposure with firearm violence or related risks were also identified. These studies addressed individual alcohol use,^31^,^58^,^63^,^64^,^69^,^71^ AUD,^60^,^67^,^71^ alcohol outlets,^71^,^73^,^75^,^76^,^78^ and alcohol control policies,^82^,^85^ and the majority of the associations were not statistically significant or had mixed statistical inferences.^31^,^58^,^59^,^63^,^64^,^66^,^69^,^71^,^73^,^75^,^76^,^78^,^84^,^85^

Studies were published in journals reflecting varying disciplines, including medicine, surgery, public health, drug and alcohol specialties, and violence specialties. The most common sources of funding were the National Institutes of Health, the Centers for Disease Control and Prevention, and university or academic medical center funds. Other sources of funding included the Veterans Administration, Social Sciences and Humanities Research Council of Canada, state governments, local governments, and foundations (Heising-Simons Foundation, Grandmothers Against Gun Violence, Fund for a Safer Future, California Wellness Foundation, National Collaborative for Gun Violence Research, Joyce Foundation, Chan Zuckerberg Biohub San Francisco, Michigan Healthy Asian Americans Endowment Fund). Notably, 10 studies stated that they had no source of funding.^50^,^52^,^55^,^57^,^61^,^62^,^65^,^68^,^72^,^84^

### Results of Individual Sources of Evidence and Synthesis of Results

#### Etiology: Individual-level alcohol use or AUD and firearm use

Appendix 1 section A describes the characteristics and results of the 21 studies examining either alcohol use or AUD in relation to firearm use.^31^,^45^–^64^ As in Branas et al. 2016, the category of firearm use included firearm ownership, access, carrying, safe storage, making threats, and perpetration of firearm assault behaviors. Findings from 11 studies suggest that across distinct populations and study designs, and controlling for various potential confounders, alcohol use was associated with greater likelihood of firearm ownership, desire to own a firearm, firearm access (have a firearm or could acquire one quickly), firearm purchases, and unsafe storage practices.^31^,^45^–^54^ These associations were observed across multiple types of alcohol use, including lifetime, past year, past 30 days, weekly, binge, chronic, and excessive drinking, and varying populations, including adult women, adolescents, veterans, Asian Americans, and general populations in the United States and Brazil. An additional five studies consistently linked past 30-day alcohol use or AUD to substantially elevated rates of firearm or handgun carrying.^55^–^59^ A final five studies showed mixed results relating alcohol use (same day, past month, past 6 months; seeking withdrawal management for AUD) to indicators of firearm use or perpetration (conflict involving a firearm;^62^,^63^ firearm assault perpetration;^64^ impaired hunting or target shooting;^61^ or an index that combined multiple measures of firearm involvement, including firearm carrying, being threatened with a firearm, and shooting another person^60^). A majority of the studies in this section were cross-sectional,^31^,^45^–^53^,^60^–^62^ but several were cohort studies relating longitudinal trajectories of alcohol use with firearm behaviors.^54^,^57^–^59^,^63^,^64^ These studies were more likely to show medium-sized statistically significant effects, particularly for handgun carrying.

#### Etiology: Individual-level alcohol use or AUD and firearm injury

Appendix 1 sections B to F describe the characteristics and results of the seven studies examining either individual-level alcohol use or AUD in relation to firearm injury or death.^65^ ^71^ The studies considered varying populations and study designs, and controlled for distinct sets of potential confounders. Only two of the studies involved longitudinal follow-up of individuals over time,^69^,^70^ and many estimates were imprecise. The current review distinguished between population-based studies that included both injured and non-injured persons,^67^–^71^ which may allow for inferences about injury prevention, and studies exclusively of decedents or injured persons,^65^,^66^ which may allow for inferences about alcohol- or firearm-involvement in injury, but not overall injury prevention.

Among studies examining outcomes related to self-harm, acute alcohol use at the time of death was positively associated with firearms being used as the means of suicide (compared to non-firearm means).^65^,^66^ Documented AUD was associated with greater risk of firearm self-harm.^70^ Regarding interpersonal firearm violence outcomes, higher state-level rates of heavy drinking were associated with higher rates of firearm homicide.^68^ Documented AUD was associated with greater risk of legal intervention injuries (i.e., those caused by law enforcement agents) and firearm homicide victimization,^69^ but lower risk of a nonfatal assault injury involving a firearm (versus non-firearm means).^67^

#### Etiology: Alcohol outlets and firearm injury or crime

Appendix 1 section G describes the characteristics and results of the eight studies examining alcohol outlets in relation to firearm injury or crime.^71^–^78^ The methods and measures used were diverse, with no two studies using identical exposure definitions or statistical methods, apart from two studies by the same team.^77^,^78^ Only one of the eight studies examined firearm self-harm as the outcome;^78^ the others examined interpersonal firearm injury or crime. Results also were highly mixed. Although some studies documented positive associations between alcohol outlets and interpersonal firearm injuries or crimes, the presence and magnitude of the association appeared to depend on the distance between the outlet and the incident, the type of outlet (e.g., liquor store, beer store, bar/tavern), the city of study (e.g., Detroit versus New Orleans), and the region within the city. Arguably the most methodologically rigorous study employed a population-based case-control design to examine the impact of momentary proximity to alcohol outlets over 3-day activity paths on risk of firearm assault injury. This study found that greater proximity to liquor stores was negatively associated with firearm assaults.^75^ The one study examining firearm self-harm showed no association with alcohol outlets.^78^

#### Etiology: Alcohol-related offenses and firearm injury or crime

Appendix 1 section H describes the characteristics and results of the two studies examining the associations of individual-level alcohol offenses with firearm injury or crime.^79^,^80^ Both studies leveraged a retrospective cohort of all persons legally purchasing handguns in California in 2001, who were followed for 13 years. These thorough studies involved complex record linkages and detailed measurement of individual-, census tract- and county-level confounders. One analysis found that alcohol-related charges accrued through arrests or the legal process prior to the date of handgun acquisition were associated with more than double the risk of subsequent firearm suicide.^79^ The other study found that people with DUI convictions before the date of first handgun purchase were at nearly three times the risk of perpetrating firearm-related violent crimes.^80^

#### Intervention: Alcohol policies and firearm injury or crime

Appendix 1 sections I and J describe the characteristics and results of the six studies that examined the impacts of policies on firearm violence-related outcomes.^81^–^86^ This category included both alcohol control policies and policies restricting access to firearms based on prior alcohol-related offenses. Five studies relied on state-level ecological designs^81^–^85^ and one involved an agent-based model simulation.^86^

Among the four studies of alcohol control policies, one analysis of National Violent Death Reporting System (NVDRS) data linked more restrictive state alcohol control policies to lower rates of firearm suicide (versus no firearm suicide) and firearm involvement in suicide (versus suicide completed with non-firearm means).^81^ A second study of nationwide death records found that more restrictive state alcohol control policies were associated with lower rates of firearm suicide but higher rates of firearm homicide.^82^ In arguably the most methodologically rigorous study, the authors applied a synthetic control approach and found that increases in state beer excise taxes were associated with lower rates of firearm homicide among individuals ages 15 to 34 years in all states except Illinois.^83^ The fourth study found that repeals of state laws banning Sunday sales of alcohol for off-premise consumption were associated with a 17% increase in firearm homicide but no change in firearm suicide.^84^

Among studies examining policies restricting access to firearms based on prior alcohol-related offenses, a state-level cross-sectional time series study found that U.S. state intoxicated-driving laws that activated federal prohibitions on firearm access were associated with 18% to 19% lower rates of firearm homicides among women compared with states that had no legal framework for prohibiting firearms after DUI convictions. However, there was no association with firearm suicide rates.^85^ A second study using simulation modeling estimated the impact of hypothetical firearm restriction policies in New York City and found that a policy disqualifying people from purchasing firearms for 5 years after an alcohol-related misdemeanor conviction would reduce population-level rates of firearm homicide by 1% and firearm suicide by 3%.^86^

## Discussion

### Summary of Evidence

This scoping review updates Branas et al.’s 2016 systematic review on the links between alcohol use and firearm violence^22^ to examine how the literature has evolved and whether stronger studies now indicate more definitive causal links between alcohol use or alcohol-related interventions or policies with firearm violence and related risks. A total of 43 studies with 70 unique findings published between 2015 and 2023 met the inclusion criteria. The included studies involved diverse data sets, study designs, and statistical methods and assessed relationships between varied dimensions of alcohol and firearms, thereby addressing an array of distinct research questions. For example, studies assessed the effects of acute alcohol intoxication on immediate risk of firearm suicide death, the effect of prior alcohol-related convictions on subsequent risk for perpetration of violent firearm crimes, and the impacts of increases in beer excise taxes on firearm homicide victimization among young adults. Compared with the studies on alcohol use and firearm violence published in 2014 and prior captured by Branas et al.,^22^ the research captured in the current review expanded notably into new dimensions of alcohol-related risks, including AUD, alcohol-related offenses, and alcohol control policies.

Regarding the question of etiology—that is, whether alcohol use causes firearm violence—findings from the studies in this review indicated a general pattern of positive associations, although some studies did find null or protective associations. Although this review characterized the set of studies seeking to answer etiologic questions, it did not evaluate whether the studies provided definitive evidence of causality, either individually or collectively. Such analyses could be better addressed in studies that focus narrowly on the relationship between a specific exposure and outcome variable. Given the diversity of alcohol- and firearm-related factors examined across this and the 2016 review, very few studies used identical definitions of exposures or outcomes, let alone the same study designs, confounders adjustment, or study populations. Therefore, differences in findings across studies do not necessarily indicate inconsistent results, but rather may be attributable to differences in the precise research questions, study populations, and approaches. Many estimates of associations were also imprecise with wide confidence intervals; in these studies, null effects could not be distinguished from large effects. Examining the combined literature included in this and the previous review, the most consistent and robust findings linked individual-level alcohol use with firearm ownership and firearm carrying among adolescents and young adults. In contrast, the estimated impacts of proximity to or density of alcohol outlets on firearm violence were inconsistent. This variation suggests that the association of alcohol outlets with firearm violence is likely specific to the place, time, context, type of outlets, nature of the proximity, and types of firearm violence outcomes. Taken together, the literature captured in the current review continues to support a causal relationship between alcohol exposure and firearm violence. However, the conclusion of the 2016 review that experimental and quasi-experimental designs could help further determine causality remains. Additionally, replication studies may increase understanding of which types of alcohol use or alcohol exposure causally affect which types of firearm-related outcomes.

The current review also addressed the question of whether any interventions on alcohol use, alcohol availability, or other alcohol-related factors can effectively prevent firearm violence or related risks. Based on the reviewed studies, the strongest available evidence suggests that prohibiting firearm access for people with a history of alcohol-related offenses may prevent violent firearm crime perpetration, firearm suicide, and firearm homicide among specific populations. This conclusion is supported by four studies with varied approaches and study populations, including two studies leveraging a large longitudinal cohort of individuals who purchased a handgun in California,^79^,^80^ one cross-sectional time series study of U.S. state policies,^85^ and one agent-based modeling study of a simulated New York City population.^86^ This policy intervention may therefore represent an important untapped prevention strategy that could be immediately pursued by federal, state, or local governments. Given the varied scope of the study populations to date (primarily people who purchased handguns in California) and variation in the exact offenses examined (alcohol-related charges accrued through arrests or the legal process; one, two, or three or more DUI convictions; alcohol-related misdemeanor convictions; or alcohol-related arrests), further replication of these findings and refinement of the highest risk offenses would better support specific policy opportunities.

Other candidate interventions include restrictions on alcohol outlets and other alcohol control policies. Eight studies examined the relationship between alcohol outlet density or proximity and firearm violence or related risks, but no studies tested interventions. Studies of policies or events that prompt changes in alcohol outlet densities, locations, or availability would better support causal inferences. Only four studies, all ecological in design, assessed the impacts of alcohol control policies on firearm violence or related risks. Two studies examined overall policy restrictiveness, one examined increases in beer excise taxes, and one examined repeals of laws banning Sunday sales of alcohol for off-premise consumption. Although the findings generally support the potential of alcohol control policies to prevent firearm suicide and homicide—with particularly strong evidence of the link between beer excise taxes and youth firearm homicide—policy decisions would be better supported by additional high-quality, individual-level longitudinal studies on the impacts of additional specific alcohol control policies. Some examples are discussed further in the next section.

Although a larger body of research has evaluated the impacts of alcohol-related exposures on violence generally, the current review was restricted to firearm violence-related outcomes. For example, much research has linked alcohol outlet density, restrictions on hours of alcohol sales, and alcohol taxation to violent crime and injury in general; however, few studies have specifically considered firearm assault or self-harm.^22^,^87^–^99^ Separate analyses of firearm-related outcomes are important because the epidemiology and causes of firearm and non-firearm violence differ dramatically. For example, disparities between Black and White populations in firearm homicide are large (with an additional 27 homicides per 100,000 population in Black populations compared with White populations) and vary strikingly across states. In contrast, corresponding annual disparities in non-firearm homicide are much smaller (about 3 additional homicides per 100,000), with minimal state variation.^100^ Violence prevention interventions can also have opposing effects on firearm compared to non-firearm assault,^101^ further supporting the need for a distinct focus.

### Research Gaps and How to Address Them

As discussed above, additional etiologic research could help clarify the impacts of specific types of alcohol use and alcohol availability (outlets). Additionally, to date, there are no published studies of the impacts of most state alcohol control policies or any local alcohol control policies on firearm violence. Unstudied policies include price controls, restrictions on hours of sale, bans on sales or promotions, age limits, licensing requirements, event-based restrictions, and home delivery policies at the federal, state, and local levels. In their 2016 review, Branas et al. concluded that: “Policies that rezone off-premise alcohol outlets, proscribe blood alcohol levels and enhance penalties for carrying or using firearms while intoxicated, and consider prior drunk driving convictions as a more precise criterion for disqualifying persons from the purchase or possession of firearms deserve further study.”^22^^(p32)^ Except for DUI convictions as firearm disqualification criteria, these areas of research remain unassessed and represent opportunities for future study.

There are several other key research gaps. First, there is a lack of research on alcohol and firearm violence examining differential associations by demographic subgroup or whether the given alcohol-related exposure exacerbates or reduces disparities in firearm violence. Social policies often have differential impacts on population subgroups, yet assessments of effect measure modification remain rare.^41^,^42^ Of the included studies, only 40% considered effect measure modification by any population characteristic, and only one study examined heterogeneity by race/ethnicity. This research gap is striking given the extreme racial/ethnic disparities in firearm violence outcomes. For example, compared with non-Hispanic White people, rates of firearm homicide victimization are 12 times higher among non-Hispanic Black people, nearly four times higher among non-Hispanic American Indian and Alaska Native people, and more than two times higher among Hispanic people.^102^ There are several reasons to expect that impacts of alcohol-related social policies may differ by race/ethnicity. For instance, the prevalence of any alcohol consumption is higher among White people;^103^ therefore, alcohol control policies may primarily benefit them, thereby exacerbating inequities. On the other hand, alcohol outlets are disproportionately located in communities of color^104^ that also experience disproportionate harms from drinking.^105^ Therefore, alcohol control policies that de-concentrate alcohol outlets or otherwise reduce the harms of alcohol misuse may reduce related inequities. Research on disparities in alcohol-related outcomes across other dimensions (e.g., gender, socioeconomic status, disability status, housing status) would also be valuable. Future research on subgroup differences in the links between alcohol exposure and firearm violence-related outcomes may help illuminate implications for inequities.

Second, only two studies examined individual-level alcohol use, AUD, or alcohol offenses in relation to firearm violence perpetration rather than victimization. This is likely because information on victims is often more accessible in common sources of data on firearm violence, such as death or hospitalization records. However, a detailed understanding of the characteristics and behaviors of perpetrators may be critical to inform prevention efforts. Prospective survey-based data collection and data linkage to records of criminal justice involvement such as that conducted by Schleimer et al.,^79^ Kagawa et al.,^80^ and others will be key to pursuing such research.

Third, the science on alcohol and firearm violence would benefit from additional randomized trials and quasi-experimental studies. Among the studies included in this review, the level of confounding control—which can be assessed crudely based on the number of confounders included—varied considerably across studies. High-quality observational studies can provide strong evidence to guide policy, but randomized and quasi-randomized designs are essential to enhance the level of confounding control. Although this review separated the question of etiology from that of intervention, interventional studies would also help to establish etiology. Given the known harms of alcohol misuse, randomization of alcohol use at the individual level is generally unethical. However, approaches that randomly stagger the rollout of local alcohol control policies could provide compelling evidence. Local policy randomization is rare, but not impossible in the presence of a strong academic-public partnership, and precedents do exist.^106^,^107^ For example, investigators could work with state officials to randomize county public health departments to early versus late rollout of a public media campaign to reduce alcohol misuse. At a minimum, additional quasi-experimental studies leveraging erratic changes in alcohol control policies as a source of variation in alcohol use could rigorously address etiology and help determine whether and which alcohol control policies are effective in preventing firearm violence.

### Additional Methodological Considerations

Although this review did not formally assess study quality or risk of bias because of the extreme heterogeneity in research questions, study populations, and methodological approaches, it was clear that study quality varied. Some studies employed cross-sectional surveys of small, non-representative populations and controlled for only a few potential confounders, whereas other studies involved large-scale individual-level longitudinal follow-up and detailed, multilevel confounder assessment and adjustment. The quality of measurement also varied considerably. For example, although the presence of AUD is ideally measured based on a diagnosis from a health care provider, the studies in this review commonly used survey-based screening tools, such as the Alcohol Use Disorders Test Version C, or secondary diagnostic codes recorded in hospitalization records based on the World Health Organization’s International Classification of Diseases. Death, injury, and crime records were the most common sources of outcome data, but each of these has recognized limitations for measuring firearm violence outcomes.^108^ Along with needed investments in firearm violence data infrastructure,^108^ studies that examine outcomes from multiple data sources may be better positioned to produce conclusive findings. Many studies were also inconclusive because of imprecise effect estimates. Future studies would benefit from study designs and statistical analysis approaches that optimize precision, for example, by leveraging the recent advances in quasi-experimental methods^109^ or Bayesian approaches that incorporate prior knowledge to enhance precision.

### Limitations

This scoping review was subject to several limitations. Given the small number of included studies relative to the great heterogeneity in research topics, study populations, and methodological approaches, it was not possible to conduct meta-analyses or systematic assessments of study quality. Systematic reviews that focus more narrowly on one specific dimension of alcohol and one specific dimension of firearms (e.g., the impact of city policies restricting alcohol outlet density on firearm assault) will be better positioned to assess study quality and quantitatively synthesize the evidence. For the same reason, it was not possible to incorporate formal assessments of publication bias.

### Conclusions

In conclusion, as firearm injuries, including homicide and suicide, continue to escalate in the United States, the role of alcohol in contributing to firearm violence and related risks remains significant. Taken together, the literature supports a causal role for alcohol exposure—including intoxication, ongoing AUD, and exposure to alcohol-concentrated environments—in contributing to firearm-related harm. This is especially alarming given that per capita, alcohol consumption in the United States has been sharply increasing for more than a decade.^110^ Concomitantly, alcohol-related mortality has increased—including by more than 25% in the first year of the COVID-19 pandemic.^111^ Reducing population-level alcohol consumption and alcohol-related harms, including firearm morbidity and mortality, across the United States is a tremendous opportunity to advance public health. Rigorous studies evaluating the impact of specific federal, state, and local alcohol control policies on firearm violence can play a pivotal role in informing this public health response.

**KEY TAKEAWAYS**
- Alcohol exposures, including alcohol use, misuse, use disorder, outlets, and policies, may be modifiable risk factors for firearm violence.
- Studies published since 2014 support a causal relationship between various alcohol exposures and firearm-related harms.
- Policies controlling the availability of alcohol and prohibiting firearm access among individuals with alcohol-related offense histories show promise for the prevention of firearm violence.
- Investigation of subgroup differences, alcohol use among perpetrators of firearm violence, policies restricting alcohol outlet density, and randomized or quasi-experimental study designs with longitudinal follow-up would further support inferences to inform prevention efforts.

## Figures and Tables

**Table 1:** Search Strategy and Results by Database

Database	Search Strategy	Number of Articles Identified
PubMed	((((“firearms”[MeSH Terms] OR “firearm”[All Fields] OR “gun”[All Fields]) AND (“ethanol”[MeSH Terms] OR “ethanol”[All Fields] OR “alcohols”[MeSH Terms] OR “alcohol”[All Fields] OR “alcohol outlet”[All Fields])) NOT “animals”[MeSH Terms:noexp]) AND 2015/01/01:2023/12/15[Date - Publication]) NOT (“meta analysis”[Publication Type] OR “review”[Publication Type] OR “systematic review”[Filter])	520
Scopus	(“firearm” OR “firearms” OR “gun” OR “guns”) AND (“ethanol” OR “alcohol” OR “alcohol outlet”), 1/1/2015–12/15/2023, NOT review articles, (articles)	448
Web of Science (SCI-EXPANDED, SSCI)	(“firearm” OR “firearms” OR “gun” OR “guns”) AND (“ethanol” OR “alcohol” OR “alcohol outlet”), 1/1/2015–12/15/2023, NOT review articles	257

**Table 2:** Number of Studies on Alcohol and Firearm Violence by Topic, for Branas et al. 2016 Review Versus the Current Review

Level of Alcohol Exposure and Topic	Branas et al. 2016 (Studies 1975 to 2014)	Current Review (Studies 2015 to 2023)
***Individual level – Alcohol use***		
Alcohol use and firearm use* (descriptive)	8 studies included alcohol use among firearm users^25^,^112^–^118^ 6 studies included firearm use among alcohol users^119^–^124^	Not covered
Alcohol use and firearm use (reporting adjusted associations)	10 studies^25^,^29^,^114^,^120^–^122^,^125^–^128^	17 studies^31^,^45^–^49^,^51^,^53^–^55^,^57^–^59^,^61^–^64^
Acute alcohol use among firearm injury decedents (descriptive)	12 studies included firearm homicide^133^–^135^,^137^,^152^,^155^–^159^ 18 studies included firearm suicide^132^–^149^ 1 study included firearm accident^133^ 4 studies included firearm death (homicide, suicide, and accident combined)^129^–^132^	Not covered
Acute alcohol use and firearms versus other means among decedents (reporting adjusted associations)	4 studies of suicide decedents^141^,^142^,^145^,^151^	2 studies of suicide decedents^65^,^66^
Alcohol use and firearm injury (reporting adjusted associations)	1 study included self-harm^139^ 1 study included interpersonal violence^152^	2 studies included interpersonal violence^68^,^71^
***Individual level – Alcohol use disorder***		
Alcohol use disorder and firearm use	0 studies	4 studies^50^,^52^,^56^,^60^
Alcohol use disorder and firearm injury	0 studies	3 studies included interpersonal violence^69^,^70^ 2 studies included self-harm^69^,^70^
***Individual level – Alcohol offenses***		
Alcohol offenses (e.g., DUI conviction) and firearm injury	0 studies	1 study included interpersonal violence^79^ 1 study included self-harm^79^
***Neighborhood level***		
Alcohol outlets and firearm injury or crime	2 studies included firearm assault or homicide^152^,^153^ 1 study included self-inflicted firearm injury and firearm suicide^139^	7 studies included interpersonal violence^71^–^77^ 1 study included self-harm^78^
***Policy level***		
State policies restricting the intersection of alcohol and firearms (descriptive)	1 study^154^	Not covered
Alcohol control policies and firearm injury or crime	0 studies	3 studies included interpersonal violence^82^–^84^ 3 studies included self-harm^81^,^82^,^84^
Alcohol-related firearm policies and firearm injury or crime	0 studies	2 studies included interpersonal violence^85^,^86^ 2 studies included self-harm^85^,^86^

*Note*:

* Firearm use includes ownership, access, carrying, storage, or threats

**Appendix 1:** Characteristics and Results of Studies Examining Alcohol-Related Exposures and Firearm-Related Outcomes

Study	Study Population	Data Source(s)	Exposure(s)	Outcome(s)	Analytic Approach	Main Finding(s)
**A. Individual-level alcohol use or alcohol use disorder and firearm use**						
Justus 2020^45^	10,250 respondents of three random cross-sectional victimization surveys conducted in São Paulo city, Brazil, in 2003, 2008, and 2013 with people ages 16 or older	São Paulo victimization surveys	Any habitual alcohol use; past month number of times consuming five or more drinks	Desire to own a firearm for protection	Cross-sectional study Probit regression reporting marginal effects at mean, adjusted for age, sex, ethnic group, immigration status, seven socioeconomic characteristics, prior crime victimization, three social capital measures, and year. EMM not considered.	People who drink alcohol, compared with people who do not drink alcohol, were 6.4 percentage points more likely (p <.01) to want a firearm even after controlling for two dozen demographic, socioeconomic, social capital, and victimization variables. There was a dose-response relationship between the number of episodes of binge drinking and the latent potential demand for firearms.
Vaddiparti 2016^46^	586 women ages 18 or older who use alcohol or drugs in St. Louis, Missouri, participating in the baseline phases of two community-based HIV prevention interventions	Sister to Sister study; Women Teaching Women study	Past 7-day binge drinking	Firearm exposure (ownership, carrying, or access)	Cross-sectional study Logistic regression adjusted for age, race, pathological gambling, lifetime cocaine dependence, major depressive disorder, and antisocial personality disorder. EMM not considered.	Binge drinking was a significant risk for gun exposure after controlling for other risk factors (OR: 1.91; 95% CI [1.19, 3.04]).
Abaya 2019^31^	2,258 adolescents ages 14 to 24 presenting to the emergency department of a freestanding children’s hospital located in a large urban area in the United States, 2013 to 2014	Survey	Lifetime alcohol use; past 30-day alcohol use	Firearm access (have a gun or could acquire one quickly)	Cross-sectional study Logistic regression adjusted for age, race, gender, trauma/assault, psychiatric concern, lifetime drug use, current drug use, substance-related impairment, physical fight, bullying (verbal, physical, cyber), depression, traumatic distress, and lifetime and current suicidality. EMM not considered.	Lifetime alcohol use was associated with 1.95 increased odds of access to a firearm compared to no access when controlling for confounders (95% CI [1.35, 2.81]). Current alcohol use was associated with 0.74 decreased odds of access to a firearm compared to no access when controlling for confounders (95% CI [0.47, 1.17]).
Rossheim 2022^47^	Sample of 183 first-time college freshmen ages 18 to 24 in 2019 at George Mason University, United States	Mason: Health Starts Here cohort study	Past 30-day binge drinking	Firearm access (have a gun or could acquire one quickly)	Cross-sectional study Logistic regression adjusted for age, sex, race/ethnicity, and marijuana use. EMM not considered.	People who engaged in binge drinking in the past 30 days (OR: 6.4; 95% CI [2.1, 19.7]) had greater odds of having rapid firearm access.
Sigel 2019^48^	Community-based sample of 1,100 adolescents ages 10 to 17 and 730 of their parents in two communities in a large, urban mountain west city in the United States at high risk for violence, July 2012 to March 2013	Baseline survey of Communities That Care (CTC) intervention study	Past-year alcohol use	Firearm access (have a gun or could acquire one quickly); Firearm possession	Cross-sectional study Logistic regression adjusted for age, violence perpetration, cyberbullying victimization, marijuana use, internalizing symptoms, peer problems, and parents with guns at home. EMM not considered.	Youth reporting past-year alcohol use had 2.02 times the odds of firearm access (95% CI [1.20, 3.80]) and 2.3 times the odds of firearm possession (95% CI [0.46, 11.8]) compared to youth without past-year alcohol use, adjusting for covariates.
Wu 2023^49^	916 self-identified Asian Americans ages 18 or older from a national sample of U.S. adults using a quota-based sampling implemented by Dynata	Dynata panel	Number of days per week of alcohol use	Firearm purchase during COVID-19 pandemic	Cross-sectional study Path analysis, probit regression adjusted for gender, age, income, education, and marital status. EMM not considered.	Alcohol use was associated with greater likelihood of purchasing a firearm (beta: 0.084; 95% CI [0.020, 0.148]).
Fischer 2023^50^	2,326 participants ages 18 or older of the 2022 National Health and Resilience in Veterans Study, a nationally representative sample of U.S. veterans	National Health and Resilience in Veterans Study	Lifetime history of alcohol use disorder	Firearm ownership	Cross-sectional study Logistic regression adjusted for age, sex, income, home ownership, residence in New England, children under age 18 living in household, metro area, political ideology, VA health care, years of military service, and eight psychiatric characteristics. EMM not considered.	U.S. military veterans reporting lifetime history of alcohol use disorder had 1.74 times the odds of firearm ownership compared to those without a history of alcohol use disorder (95% CI [1.43, 2.11]).
Morgan 2018^51^	State representative samples of 34,884 noninstitutionalized adults ages 18 or older in Washington state, 2013 to 2016	Washington State BRFSS	Binge drinking; chronic alcohol use	Household firearm ownership; household firearm safe storage	Cross-sectional study Poisson regression, adjusted for age, race/ethnicity, gender, income, education, employment, marital status, and urbanicity. EMM not considered.	Binge and chronic alcohol use were somewhat more prevalent among adults from households that owned firearms (PR: 1.2; 95% CI [1.1, 1.3]; PR: 1.2; 95% CI [1.1, 1.4], respectively) and among those living in households not practicing safe storage (PR: 1.4; 95% CI [1.2, 1.7]; PR: 1.9; 95% CI [1.5, 2.3], respectively).
Smith 2020^52^	Sample of 201 military service members and veterans ages 18 to 60 recruited from individuals using GI Bill educational benefits September to December 2015	Survey	Potential alcohol misuse (AUDIT-C total score)	Handgun ownership; long gun ownership; inventory of six firearm storage practices	Cross-sectional study MANOVA adjusted for fearlessness about death, depression symptoms, thwarted belongingness, perceived burdensomeness, and PTSD symptoms. EMM not considered.	Individuals reporting greater potential alcohol misuse symptoms were those endorsing or declining to respond to handgun ownership (versus those who denied handgun ownership; F = 6.17; p = 0.003), those endorsing or declining to respond to long gun ownership (versus those who denied long gun ownership; F = 3.40; p = 0.036), and those reporting that they did (versus did not) store their firearms loaded (F = 5.88; p = 0.016) and unlocked (F = 9.61; p = 0.002).
Morgan 2019^53^	1,756 respondent households of the 2013 and 2016 Washington state BRFSS (random sample from noninstitutionalized adult population ages 18 or over) that reported owning a firearm	Washington State BRFSS	Adult in household reported binge, chronic, or excessive drinking	Adult in household reported storing one or more firearms unsafely (i.e., not locked or loaded)	Cross-sectional study Poisson regression adjusted for child age; race/ethnicity; annual household income; urbanicity; adult respondent’s age, sex, and marital status. Considered EMM by age and urbanicity.	Firearms were more likely to be stored unsafely in homes in which an adult reported alcohol misuse (PR: 1.20; 95% CI [1.07, 1.35]).
Martin-Storey 2018^54^	650 survey respondent families in which parents reported owning firearms but did not keep them stored in a locked cabinet, from a U.S. nationally representative sample of children born in 2001	Early Childhood Longitudinal Study-Birth Cohort 2003	Parental average number of drinks per week	Firearm safe storage trajectory (locked and unloaded)	Cohort study Logistic regression adjusted for maternal race/ethnicity, education, depression; male partner cohabitation; siblings in the home, household income, residential move, perceived neighborhood safety, urbanicity, region, state firearm household ownership rate, and state violent crime rate. EMM not considered.	Each additional increase in the level of parental drinking was associated with 18% lower odds (95% CI [0.72, 0.94]) of moving to a safer firearm storage strategy.
Khubchandani 2018^55^	U.S. nationally representative samples of 22,741 African American students in grades 9 to 12 attending public and private schools in the United States who were respondents of YRBS surveys, 2001 to 2015	YRBS	Past-month alcohol use on school property	Past 30-day firearm carrying	Cross-sectional time series Logistic regression adjusted for tobacco and drug use behaviors, academic and lifestyle behaviors, injury and violent risk behaviors, and psychosocial risk behaviors. EMM not considered.	Alcohol use (one drink or more) at school was associated with increased odds of gun carrying among African American females (OR: 9.18; 95% CI [6.27, 11.70]) and males (OR: 10.48; 95% CI [8.54, 12.87]).
Kagawa 2019^56^	U.S. nationally representative sample of 10,123 adolescents ages 13 to 18	NCS-A	Alcohol use disorder according to WHO CIDI	Past 30-day firearm carrying	Case-control study Poisson regression adjusted for sex, age, race/ethnicity, family income, parent education, number of parents in the home, region of the county, and urbanicity. EMM not considered.	The prevalence of gun carrying was greater among adolescents with alcohol use disorder (adjusted PR: 1.83; 95% CI [0.97, 3.47]).
Dong 2019^57^	1,574 urban youth ages 16 to 28 from a U.S. nationally representative sample who reported carrying a handgun at least once between 1997 and 2011	NLSY97	Past 30-day alcohol use	Handgun carrying trajectory, based on reported handgun carrying at each survey wave	Cohort study Multinomial logistic regression, adjusted for baseline gender, race/ethnicity, region, victim of repeated bullying before age 12, and exposure to gun violence before age 12, and for time-varying poor mental health, marijuana use, hard drug use, police arrest, gang in neighborhood or school, income, and age. Considered EMM by age.	During emerging adulthood (ages 20 to 24), alcohol use was associated with a higher risk of being in the bell-shaped firearm-carrying group when compared to the declining group (RRR: 1.40; 95% CI [1.04, 1.89]). As compared to the declining group, alcohol use characterized the late-initiating group (RRR:1.76; 95% CI [1.21, 2.54]), and the high-persistent firearm-carrying group (RRR:1.66; 95% CI [1.03, 2.67]). During young established adulthood (ages 24 to 28), alcohol use was associated with a higher risk of being in the late-initiating group (RRR:1.55; 95% CI [1.08, 2.22]).
Dong 2021^58^	U.S. nationally representative sample of 6,748 youth born between 1980 and 1984	NLSY97	Alcohol use longitudinal trajectory, based on past 30-day alcohol use at each survey wave	Handgun carrying trajectory, based on reported handgun carrying at each survey wave	Cohort study Multinomial logistic regression adjusted for smoking trajectory, marijuana use trajectory, hard drug use trajectory, race, gender, age, region, income, urbanicity, poor mental health, gang nearby, police arrest, bullying victimization, gun violence exposure, and violent victimization. EMM not considered.	The risk of being in the declining trajectory of handgun carrying (compared with very-low trajectory) was higher for participants who were in the decreasing or medium-decreasing trajectories of drinking (RRR:1.94, 95% CI [1.30, 2.89]) and lower for those who were in the increasing (RRR: 0.62, 95% CI [0.40, 0.95]) trajectory of drinking.
Ellyson 2023^59^	2,002 adolescents and young adults ages 12 to 26 growing up in 12 rural communities in seven states with surveys collected 2004 to 2019	Community Youth Development Study	No alcohol use in past 30 days; alcohol use in past 30 days but without five or more drinks in a row in past 2 weeks; five or more drinks in a row in past 2 weeks	Past-year handgun carrying	Cohort study Logistic regression with GEE adjusted for individual-level demographic characteristics, individual/peer-level risk and protective factors, family-level risk and protective factors, community demographic characteristics, and community-level risk and protective factors. Considered EMM by age.	During adolescence (ages 12 to 18), those who drank heavily had 1.43 times the odds (95% CI [1.01, 2.03]) of subsequent handgun carrying compared to those who did not drink alcohol, and those who consumed alcohol but did not drink heavily had 1.30 times the odds of subsequent handgun carrying compared to those who did not drink alcohol (95% CI [0.98, 1.71]). During young adulthood (ages 19 to 26), associations of alcohol use (OR: 1.28; 95% CI [0.94, 1.63]) and heavy drinking (OR: 1.38; 95% CI [1.08, 1.68]) were similar to adolescence.
Stein 2018^60^	Sample of 386 people entering a brief, inpatient opioid detox and 51 people seeking alcohol detox at the same facility in Massachusetts between October 2016 and April 2017	Survey	Seeking withdrawal management for AUD versus opioid use disorder	10-item index of firearm involvement, including carrying, being threatened, and shooting another person	Cross-sectional study Negative binomial regression adjusted for age, sex, race, ethnicity, years of education, employment status, homelessness, current legal status, and prior history of incarceration. EMM not considered.	People who misused opioids had significantly higher rates of gun involvement than people in alcohol detoxification (OR for item endorsement on 10-item gun involvement index: 2.15; 95% CI [1.58, 2.96]).
Nobles 2020^61^	Random sample of 2,349 students ages 17 and older in 40 courses at a large public university in the Southeastern United States responding to a behavioral health survey, 2012	Survey	Past month number of days of alcohol use	Impaired firearm use: Used a firearm or hunted with a firearm after consuming alcohol (five or more drinks)	Cross-sectional study Logistic regression adjusted for age, gender, race, income, employment, family income, fraternity/sorority, low self-control, peer substance use, perceived stress, marijuana use, and substance use-related arrest. EMM not considered.	Frequency of alcohol use was associated with a greater odds of impaired firearm use (OR: 1.36; not statistically significant).
Terribele 2021^62^	Brazil nationally representative sample of 102,072 9th grade students from public and private schools, 2015	Brazil National School Health Survey (PeNSE)	Past-month alcohol use	Past 30-day fight involving a firearm	Cross-sectional study Poisson regression, adjusted for region, public versus private school, age, skin color/race, maternal schooling, cohabitation, and illicit drug use. Considered EMM by gender.	Boys and girls consuming alcohol in the past month had 2.83 (95% CI [2.45, 3.27]) and 2.37 (95% CI [1.89, 2.97]) times the prevalence of exposure to firearm violence, respectively, adjusting for covariates.
Carter 2017^63^	Sample of 349 youth ages 14 to 24 who used drugs seeking emergency department care for assault injury, and a proportionally sampled comparison group of 250 youth who used drugs presenting for non-assault injury reasons, recruited from the Hurley Medical Center in Flint, Michigan	Flint Youth Injury (FYI) Study	Alcohol use within 3 hours prior to the conflict; alcohol use at any time during that day	Conflict event involving a firearm versus conflict event without firearm involvement; day that involved a firearm conflict versus not (among those reporting any firearm conflict)	Cohort study Logistic regression of event-level and day-level data. The event-level analysis adjusted for sex, race, age, public assistance, assault injury at baseline, community violence exposure, gun possession, drug use disorder, PTSD, marijuana use, and reason for conflict. The day-level analysis adjusted for age, sex, race, public assistance, assault injury at baseline, community violence exposure, and illicit drug use. EMM not considered.	Alcohol use in the 3 hours prior to conflict was not associated with the likelihood that a non-partner conflict involved firearms (OR: 0.68; 95% CI [0.37, 1.25]). Among the subsample of youth reporting non-partner firearm conflicts, alcohol use was associated with greater odds that a day involved a non-partner firearm conflict versus a non-conflict day (OR: 2.80; 95% CI [1.87, 4.17]).
Goldstick 2019^64^	Sample of 123 youth ages 14 to 24 who used drugs recruited from the Hurley Medical Center in Flint, Michigan, 2009 to 2016	Flint Youth Injury (FYI) Study	Past 6-month alcohol use	Transitions into and out of firearm assault perpetration behaviors	Cohort study Markov chain models adjusted for age, gender, race, non-firearm partner and non-firearm non-partner aggression, gun violence victimization, community violence exposure, friend negative influence, retaliatory attitudes, and marijuana use. EMM not considered.	Alcohol use did not have an effect on the rate of transition into firearm assault behavior (HR: 0.99; 95% CI [0.96, 1.03]) or out of firearm assault behavior (HR: 1.01; 95% CI [0.97, 1.05]).
**B. Alcohol use and firearms versus other means among decedents/injured persons**						
Choi 2018^65^	All suicide decedents age 50 and over captured in the U.S. NVDRS, 2005 to 2015 with alcohol test results (*n =* 29,115)	NVDRS	BAC > 0.0 at time of death; BAC > 0.08 at time of death	Firearm as means of suicide (versus other means)	Cross-sectional study Logistic regression adjusting for age, gender, race, marital status, education, military status, and 11 potentially precipitating risk factors (e.g., depressed mood at time of injury). EMM not considered.	Use of a firearm as the means of suicide was associated with greater odds of BAC > 0 at death (OR: 1.02, 95% CI [0.93, 1.12]) and greater odds of BAC > 0.08 at death (OR: 1.85, 95% CI [1.59, 2.16]).
Kim 2023^66^	All 148,823 suicide decedents ages 18 and over captured in NVDRS 2003 to 2020 with BAC test results	NVDRS	BAC > 0.08 at time of death	Firearm as means of suicide (versus other means)	Cross-sectional study Logistic regression adjusted for marital status, education, and race/ethnicity. Considered EMM by age and sex.	Alcohol intoxication was associated with using a firearm as the method of suicide for young (ages 18 to 34; RR: 1.31; 95% CI [1.22, 1.40]) and middle-aged (ages 35 to 64; RR: 1.34; 95% CI [1.27, 1.39]) females but not older females (ages 65 and over; RR: 1.01; 95% CI [0.87, 1.17]). Among males, the association was significant for all age groups (young: RR: 1.28; 95% CI [1.25, 1.30]; middle-aged: RR: 1.17; 95% CI [1.15, 1.19]; older: RR: 1.04; 95% CI [1.01, 1.07]).
**C. Alcohol use disorder and firearms versus other means among decedents/injured persons**						
Lovelady 2022^67^	1,541 Black men ages 18 to 44 with assault injuries discharged from Alaska hospitals, 2005 to 2014	Alaska statewide hospital discharge data	AUD ICD-9 diagnosis in hospital records at time of injury	Firearm as means of assault injury (versus other means)	Cross-sectional study Logistic regression adjusted for age, marital status, region, source of insurance payment, assault death, admission week day, episodic mood disorder, and schizophrenic disorder. EMM not considered.	Documented alcohol use disorder was associated with lower odds of assault admission due to firearms (OR: 0.479; 95% CI [0.317, 0.722]).
**D. Alcohol use and firearm injury**						
Scantling 2022^68^	All people in the United States, 2013 to 2016	CDC WONDER, FBI UCR, BRFSS	Prevalence of heavy drinking (14 drinks or more per week for men or seven drinks or more per week for women)	Firearm homicide rate	State-level cross-sectional time series Linear regression with state fixed effects. EMM not considered.	A 1-unit increase in the prevalence of heavy drinking was associated with a 0.223 higher rate of firearm homicide per 100,000 (95% CI [0.016, 0.450]).
**E. Alcohol use disorder and firearm injury**						
Mills 2018^69^	763 firearm injury cases and 335 controls ages 13 and older who were unintentionally injured motor vehicle collision passengers in Seattle, Washington, 2010 to 2014	Harborview Medical Center trauma registry, Washington state death records	Alcohol use-related diagnoses recorded in hospital record ICD-9 codes in 2 years prior to injury	Firearm assault, self-harm, and legal intervention injury (versus motor vehicle collision injury)	Case-control study Multinomial logistic regression adjusted for age, gender, race, and arrest history. EMM not considered.	The legal intervention firearm injury cases were more likely than controls to have diagnoses involving alcohol (OR: 4.06, 95% CI [1.04, 15.84]), but assault firearm injury cases (OR: 0.64; 95% CI [0.20, 2.03]) or self-harm firearm injury cases (OR: 1.66; 95% CI [0.63, 4.36]) were not.
Schleimer 2023^70^	All people who purchased a handgun legally in California who died between 2008 and 2013 of firearm suicide (*n =* 3,862) or unintentional motor vehicle crashes (*n =* 1,554)	California Department of Justice Dealer’s Record of Sale database, California state death records, California statewide emergency department and hospital discharge data	AUD or alcohol poisoning ICD-9 diagnosis in emergency department or hospital records in 3 years prior to death	Firearm suicide versus unintentional motor vehicle crash death	Case-control study Logistic regression adjusted for sex, age, calendar year, marital status, educational attainment, urbanicity, suicidal ideation/attempt, mental illness, drug use disorder/poisoning, pain, chronic disease, and prior assault injury. EMM not considered.	Odds of death by firearm suicide versus motor vehicle crash was 1.06 times higher for decedents with an AUD or alcohol poisoning diagnosis in the 3 years prior to death versus those without such a diagnosis, adjusting for covariates (95% CI [0.80, 1.40]).
**F. Multi-level alcohol use or exposure and firearm injury**						
Hohl 2017^71^	All 161 people ages 13 to 20 who were homicide victims in Philadelphia, Pennsylvania, matched to 172 randomly selected controls from the general population, 2010 to 2012	Philadelphia police and medical examiner reports	BAC > 0.0 at time of death; history of alcohol misuse; caregiver alcohol misuse; alcohol outlets density; visibility of bars/taverns, beer/corner stores, or alcohol ads	Firearm homicide	Case-control study Conditional logistic regression, matched on sex, hour of the day, and indoor/outdoor status, and adjusted for age, race, school suspensions, prior arrest, and neighborhood percentage Hispanic. EMM not considered.	Adolescents with a history of alcohol misuse (OR: 4.1; 95% CI [1.2, 14.0]) or living in neighborhoods with high densities of alcohol outlets (OR: 3.2; 95% CI [1.1, 9.1]) had increased odds of firearm homicide. Firearm homicide was not significantly associated with adolescent alcohol use at the time of the event, caregiver alcohol misuse, or visibility of alcohol outlets or advertisements.
**G. Alcohol outlets and firearm injury or crime**						
Oliphant 2021^72^	1,020 geolocated fatal and nonfatal shooting incidents with at least one confirmed victim in Detroit, Michigan, in 2020, combined with point data on place-based characteristics of communities (e.g., alcohol outlets, schools)	Detroit Police Department-reported shootings, 2020; alcohol outlet license listings from Michigan Department of Licensing and Regulatory Affairs	Proximity to alcohol outlets with active licenses as of February 2021	Fatal or nonfatal shooting with at least one confirmed victim	Geospatial study G-function and Cross-K Function adjusted for independent spatial clustering of alcohol outlets and shootings. Considered EMM by time of day.	Approximately 7.8 and 4.0 times as many shootings occurred within 100 feet and 200 feet of alcohol outlets, respectively, as would be expected if the locations of alcohol outlets and shootings were spatially independent.
Muggy 2022^73^	14,141 violent firearm crimes in Detroit, Michigan, 2014 to 2016, and 7,656 violent firearm crimes in New Orleans, Louisiana, 2015 to 2017	New Orleans and Detroit Police Department-reported crimes with a firearm	Proximity to LLs or alcohol outlets	Violent firearm crime incident	Geospatial study Monte Carlo simulation using Network Cross-K Function for Stochastic Spatial Events on street networks, adjusted for neighborhood SES. Considered EMM by SES index.	In Detroit, LLs were not associated with firearm crimes from 0 to 250 feet, but were negatively associated with firearm incidents from 250 to 750 feet. In New Orleans, alcohol outlets were positively associated with firearm crimes from 0 to 250 feet and from 675 to 1,000 feet, but were neutral or slightly negatively associated from 250 to 675 feet.
Jay 2020^74^	Philadelphia, Pennsylvania, city blocks with shootings, matched with similar-looking blocks with no shootings, 2017 to 2018	Philadelphia Police Department-reported shootings	Proximity to beer stores and bars/restaurants (same block, within one block, within two blocks)	Fatal or nonfatal shooting	Case-control study of matched city blocks Logistic regression adjusted for land use, demographic composition, and illegal drug activity. EMM not considered.	The fully adjusted model estimated an increased shootings risk for locations with beer stores within one block (OR: 1.5; 95% CI [1.1, 2.1]) and locations with bars/restaurants on the same block (OR: 1.6; 95% CI [1.1, 2.4]).
Morrison 2017^75^	Adolescents ages 10 to 24 presenting to the emergency department of the Hospital of the University of Pennsylvania or the Children’s Hospital of Philadelphia for a non-gun assault (*n =* 194) or gun assault (*n =* 135), and age-matched controls (*n =* 274) selected using random-digit dialing of the hospitals’ catchment areas	Space-Time Adolescent Risk Study (STARS)	Momentary proximity to alcohol outlets (bars/restaurants, beer stores, liquor stores) over 3-day activity paths	Firearm assault injury	Case-control study Conditional logistic regression adjusted for age, weekend day, context of assault (with adult family member, with peer, at home, in vehicle, on foot, other transport, possessed alcohol); and neighborhood connectedness, income, vacancy/vandalism /violence, emergency services, racial/ethnic composition, commercial land use, population density, and school density. Considered EMM by time of day.	Gun assaults were negatively associated with greater proximity to liquor stores (OR: 0.723, 95% CI [0.622, 0.841]). There was no association for bars and restaurants or beer stores.
Crandall 2015^76^	All individuals with assault GSWs that presented to trauma centers in Chicago, Illinois, 1999 to 2009	Illinois state trauma registry	LLs per Census tract	Firearm assault injury	Geospatial study of point patterns Logistic regression adjusted for race, gender, vacant housing, social security income, and estimated value of owner-occupied homes. Considered EMM by outlet type (packaged goods versus tavern).	No association between LLs and GSWs was identified for the city overall (OR: 0.97; 95% CI [0.96, 0.99]). However, five distinct regions of influence between LLs and GSWs were found. In regions with the highest association, likelihood of a GSW near a packaged LL was extraordinarily high (OR: 518.08; 95% CI [10.23, 1,000]), and tavern LLs were also very significant (OR: 21.51; 95% CI [1.81, 255.53]).
Hohl 2017^71^	All 161 people ages 13 to 20 who were homicide victims in Philadelphia, Pennsylvania, matched to 172 randomly selected controls from the general population, 2010 to 2012	Philadelphia police and medical examiner reports	BAC > 0.0 at the time of death; history of alcohol misuse; caregiver alcohol misuse; alcohol outlets density; visibility of bars/taverns, beer/corner stores, or alcohol ads	Firearm homicide	Case-control study Conditional logistic regression, matched on sex, hour of the day, and indoor/outdoor status, and adjusted for age, race, school suspensions, prior arrest, and neighborhood percentage Hispanic. EMM not considered.	Adolescents with a history of alcohol misuse (OR: 4.1; 95% CI [1.2, 14.0]) or living in neighborhoods with high densities of alcohol outlets (OR: 3.2; 95% CI [1.1, 9.1]) had increased odds of firearm homicide. Firearm homicide was not significantly associated with adolescent alcohol use at the time of the event, caregiver alcohol misuse, or visibility of alcohol outlets or advertisements.
Pear 2023^77^	All 67,850 fatal and nonfatal firearm assault injuries in California between January 2005 and September 2015 and a matched sample of 268,122 community-based controls	California state death records, California statewide emergency department and hospital discharge data	Annual population-based ZCTA-level densities of off-premise and bar/pub alcohol outlets	Firearm assault injury (fatal or nonfatal)	Case-control study g-computation with logistic regression adjusted for year; individual age, race/ethnicity, sex; ZCTA-level age, sex, and racial/ethnic composition, urbanicity, income, education, unemployment, vacant housing, business establishment density; county non-firearm violent crime rate, and property crime rate. Considered EMM by fatal versus nonfatal outcome, demographic risk group, and firearm dealer density.	Observed (versus low) densities of off-premises alcohol outlets were associated with elevated monthly risk of firearm assault per 100,000 people (RD: 0.01; 95% CI [0.01, 0.03]), but bar/pub density was not.
Pear 2023^78^	All California residents ages 10 or older, 2005 to 2015	California state death records, California statewide emergency department and hospital discharge data	Annual population-based ZCTA-level densities of off-premise and bar/pub alcohol outlets, moving weighted average for 12 months prior to case or control	Firearm self-harm injury (fatal or nonfatal)	Case-control study g-computation with logistic regression, adjusted for age, race/ethnicity, sex; ZCTA-level % ages 55 or older, % White, % male, urbanicity, median household income, % ages 25 or older with at least a bachelor’s degree, unemployment rate, spatially lagged exposure variable; year, cooler versus warmer months; indicator of 2014 or later. Considered EMM by demographic risk group and firearm dealer density.	Neither off-premise alcohol outlet density nor bar/pub outlet density was associated with firearm self-harm after adjusting for covariates.
**H. Individual-level alcohol offenses or policies restricting access to firearms based on alcohol offenses and firearm injury or crime**						
Schleimer 2021^79^	All men who legally purchased a handgun in California in 2001 and who were ages 21 or older at the time of acquisition (*n =* 101,377)	California Department of Justice Dealer’s Record of Sale database, California state death records, California Department of Justice Criminal History Information System	Alcohol charges accrued through arrests or the legal process versus neither drug nor alcohol charges, occurring on or after January 1, 1990, and before the date of handgun acquisition in 2001	Firearm suicide	Cohort study Cox proportional hazards regression adjusted for other charges and convictions, age, race, first-time purchaser; Census tract population density, age, sex and racial/ethnic distribution, SES index, alcohol outlet density; and county population size, violent and property crime rates, suicide rates, and proportion of suicides completed with a firearm. Considered EMM by characteristics of firearm purchase and offense history.	Compared with those with neither alcohol nor drug charges, those with alcohol charges had 2.22 times the hazard of firearm suicide (95% CI [1.36, 3.62]). Risk was most elevated among those with more recent charges and those with two or more charges, and in the time period closest to the purchase.
Kagawa 2020^80^	All people who legally purchased a handgun in California in 2001 who were ages 21 to 49 at the time of acquisition (*n =* 79,678)	California Department of Justice Dealer’s Record of Sale database, California Department of Justice Criminal History Information System	DUI conviction before the date of first handgun purchase in 2001	Arrest for firearm-related violent crime	Cohort study Cox proportional hazards regression adjusted for sex, age, race/ethnicity, number of prior handguns, time between most recent arrests and index purchase; Census tract population, population density, age, sex, racial/ethnic distribution, alcohol outlet density, and SES index; and county population, violent and property crime rates, and % firearm suicides. Considered EMM by race/ethnicity and sex.	Compared with purchasers who had no prior criminal history, those with prior DUI convictions and no other criminal history were at increased risk of firearm-related violent crime (adjusted HR: 2.8; 95% CI [1.3, 6.4]).
**I. Alcohol control laws and firearm injury or crime**						
Coleman 2021^81^	All suicides in 22 states covered by the 2015 U.S. NVDRS with > 30% reporting of alcohol test results	NVDRS	Restrictiveness of state alcohol policy (Alcohol Policy Scale based on 29 individual policies)	Rate of firearm suicides; firearm as means of suicide (versus other means)	Cross-sectional study State-level Poisson regression and individual-level logistic regression with GEE. Adjusted for state % male, racial/ethnic composition, % ages 21 or older, % with college degree, household income, unemployment, police officers per capita, urbanization, and religiosity. Individual-level analysis also adjusted for decedent age, sex, marital status, race/ethnicity, and mental health status. Considered EMM by firearm laws.	Higher alcohol law scores were associated with lower incidence rates of firearm suicides (IRR: 0.68; 95% CI [0.55, 0.84]), suicides involving alcohol and firearms (IRR: 0.48, 95% CI [0.35, 0.66]), and lower odds that a suicide involved firearms (OR: 0.62; 95% CI [0.47, 0.81]).
Choi 2020^82^	All people in all 50 U.S. states, 2012 to 2016	CDC WISQARS	State “alcohol freedom” (Cato Institute)	Firearm homicide rate; firearm suicide rate	Cross-sectional time series Linear regression adjusted for primary care provider rate, psychiatrist rate, poverty rate, hunting license rate, density, Medicaid generosity, worker index, violent crime prohibition laws, year, and census division. EMM not considered.	Higher levels of alcohol regulations were associated with a higher firearm-related homicide rate (RD = 0.28, SE = 0.08) and a lower firearm-related suicide rate (RD = −0.43, SE = 0.10).
Tessler 2019^83^	U.S. individuals ages 15 to 34, 2003 to 2015, except states that changed their beer excise tax but for which more than 2 years of pre-exposure data were available	CDC WONDER; APIS	Increase in state beer excise tax in Illinois (2009), New York (2009), North Carolina (2009), Connecticut (2011), and Rhode Island (2013) ranging from 10% to 27%	Firearm homicide rate among individuals ages 15 to 34	Quasi-experiment Synthetic control adjusted for % ages 18 to 24, % male, % Hispanic, % Black, % suicides with a firearm, violent crime rate, % urban, Gini coefficient, % high school graduates, % in poverty, median household income, and rates of pre-treatment firearm homicide. Considered EMM by U.S. state/size of tax increase.	The increase in beer excise tax was associated with a lower average annual firearm homicide rate among individuals ages 15 to 34 in all states except Illinois (incidence rate differences per 100,000 population: Rhode Island: 2.48, Connecticut: 2.57, New York: 1.45, North Carolina: 0.45, Illinois: 1.54).
Nicosia 2023^84^	All people in the United States, 1990 to 2019	NVSS Multiple COD Microdata; APIS	Repeal of state laws banning Sunday sales of alcohol beverages for off-premises consumption	Firearm suicide; firearm homicide	Cross-sectional time series Poisson regression adjusted for household gun ownership, income, political party control, unemployment, % ages 15 to 29, % Black, % Hispanic, education, and religion. Considered EMM by day of week.	Repealing Sunday bans is associated with an increase in firearm homicides (IRR: 1.17; 95% CI [1.03, 1.33]) but not firearm suicides (IRR: 1.03; 95% CI [0.99, 1.07]).
**J. Alcohol-related firearm laws and firearm injury or crime**						
Tessler 2022^85^	All people in all 50 U.S. states, 2013 to 2017	CDC Vital Statistics program	U.S. state intoxicated driving laws that activate federal firearm prohibitions	Firearm homicide rate; firearm suicide rate	Cross-sectional time series Negative binomial regression adjusted for age (% ages 20 or younger, 20 to 29, 30 to 39, and 40 to 49), % African American, % Hispanic, % rural, per capita income, poverty, unemployment, violent crime, rate of household firearm ownership, and state firearm laws. Considered EMM by sex.	The firearm homicide rate was 19% lower among women in states where federal firearm restrictions occurred after one to two DUI offences (IRR: 0.81; 95% CI [0.64, 1.01]) and 18% lower in states with firearm prohibitions after three or more offences (IRR: 0.82; 95% CI [0.71, 0.95]) compared with the states with no legal framework for prohibiting firearms after DUI convictions. There was no association between number of DUI activations and firearm suicide.
Cerdá 2022^86^	Simulated sample of 15% of New York City adult population (*N =* 800,000)	N/A	Hypothetical policies disqualifying those with alcohol-related misdemeanors or arrests from purchasing a firearm	Firearm homicide rate; firearm suicide rate	Agent-based model simulation. EMM not considered.	Disqualification from purchasing firearms for 5 years after an alcohol-related misdemeanor conviction reduced population-level rates of firearm homicide by 1.0% (95% CI [0.4%, 1.6%]) and firearm suicide by 3.0% (95% CI [1.9%, 4.0%]).

Legend: One “drink” refers to consumption of 1 U.S. standard serving of alcohol, equivalent to 12 ounces of regular beer, 5 ounces of wine, 1.5 ounces of distilled spirits, or 14 grams of pure alcohol. “Alcohol use” refers to one or more drinks over the designated time period. “Binge drinking” refers to four or more drinks in one session for women and five or more drinks in one session for men, with the definition of a session varying across studies. “Heavy drinking” refers to 14 or more drinks per week for men or seven or more drinks per week for women. “Chronic” or “excessive” alcohol use refers to eight or more drinks in 1 week for women and 15 or more drinks in 1 week for men. “Habitual” alcohol use refers to an affirmative answer to the question “Do you consume alcoholic beverages habitually, even if only very seldom or on special occasions?”.

*Notes*: APIS, National Institute on Alcohol Abuse and Alcoholism’s Alcohol Policy Information System; AUD, alcohol use disorder; AUDIT-C: Alcohol Use Disorders Identification Test Version C; BAC, blood alcohol content in g/dL; BRFSS, Behavioral Risk Factor Surveillance Survey; CDC, Centers for Disease Control and Prevention; CI, confidence interval; COD, cause of death; DUI, driving under the influence of alcohol; EMM, effect measure modification; FBI, Federal Bureau of Investigation; GEE, generalized estimating equations; HR, hazard ratio; ICD, International Classification of Diseases; IRR, incidence rate ratio; NCS-A, National Comorbidity Survey of Adolescents; NLSY97, National Longitudinal Survey of Youth 1997; NVDRS, National Violent Death Reporting System; NVSS, National Vital Statistics System; OR, odds ratio; PR, prevalence ratio; PTSD, post-traumatic stress disorder; RD, risk or rate difference; RR, relative risk; RRR, ratio of risk ratios; SE, standard error; SES, socioeconomic status; UCR, Uniform Crime Reports; WISQARS, Web-based Injury Statistics Query And Reporting System; WONDER, Wide-ranging Online Database for Epidemiologic Research; WHO CIDI, World Health Organization’s Composite International Diagnostics Interview; YRBS, Youth Risk Behavior Survey; ZCTA, ZIP code Census Tabulation Area.
